# PReS-FINAL-2298: Biometrics of spleen and liver in patients with childhood-onset systemic lupus erythematosus

**DOI:** 10.1186/1546-0096-11-S2-P288

**Published:** 2013-12-05

**Authors:** LM Campos, MF Silva, PF Tassetano, AG Alves, SM Rocha, M Valente, CA Silva

**Affiliations:** 1 Paediatric Rheumatology Unit, Children's Institute, University of São Paulo, São Paulo, Brazil; 2 Paediatric Radiology Unit, Children's Institute, University of São Paulo, São Paulo, Brazil

## Introduction

Involvement of the reticuloendothelial system occurs in 20-50% of patients with childhood-onset systemic lupus erythematosus (C-SLE) at disease onset. However, a systematic evaluation of liver and spleen sizes has never been performed in a pediatric population with lupus.

## Objectives

To evaluate the spleen and liver measures in C-SLE patients and to assess possible associations between reduced spleen size with demographic data, clinical features, disease activity, cumulative damage and treatment.

## Methods

Twenty four consecutive patients with C-SLE (ACR criteria) followed at the Pediatric Rheumatology Unit of Instituto da Criança HC-FMUSP underwent abdomen sonography to evaluate hepatic and splenic biometrics. The sonographic scanner used was Esaote MyLab 80 with 3-8 MHz convex transducers. The measure of liver and spleen were obtained with the patient supine and pulmonary overexpansion. Liver measure obtained was the craniocaudal diameter of the anterior portion of the right lobe in the midclavicular line, whereas splenic size was quantified through its longitudinal size. Radiologist was blind to disease characteristics. Demographic data, clinical manifestations, disease activity (SLEDAI-2K), cumulative damage (SLICC/ACR-DI) and treatment were also evaluated. Statistical analyzes were performed with the Fisher exact test and Mann-Whitney.

## Results

Splenomegaly was observed in 2 (8%), reduced spleen size in 5 (21%) and normal spleen in 17 (71%). Male gender was significantly higher in patients with low compared with normal spleen size (60% vs. 6%, p = 0.024), as well as higher median disease duration [8.8 (3-13) vs. 2 (0.4 to 7.4) years, p = 0.01] and current age [16 (14.8-17.5) vs. 13.5 (8.9-18) years, p = 0.037]. However, there was no statistical difference between the other parameters (age of onset, weight, height, and mucocutaneous, articular, serositis, hematologic, renal and neuropsychiatric involvements) assessed in C-SLE patients with low *versus *normal spleen size (p > 0.05). SLEDAI-2K scores and SLICC/ACR-DI and treatment were also comparable in both groups (p > 0.05). Furthermore, only 1 (4%) C-SLE patient had hepatomegaly and 23 (96%) normal liver size. The same patient had moderate hepatosplenomegaly with nephrotic syndrome and the SLEDAI-2K was 10.

## Conclusion

Reduced spleen size occurred in male pediatric lupus patient with long disease duration, suggesting the possibility of autosplenectomy. Future studies evaluating the splenic function, including a healthy control group, will be necessary. Nevertheless, either splenomegaly or hepatomegaly associated with disease activity was rarely observed.

## Disclosure of interest

None declared.

